# Enantiomeric-Enriched Ferrocenes: Synthesis, Chiral Resolution, and Mathematic Evaluation of CD-chiral Selector Energies with Ferrocene-Conjugates

**DOI:** 10.3390/molecules22091410

**Published:** 2017-08-25

**Authors:** Lubov V. Snegur, Yurii A. Borisov, Yuliya V. Kuzmenko, Vadim A. Davankov, Mikhail M. Ilyin, Mikhail M. Ilyin, Dmitry E. Arhipov, Alexander A. Korlyukov, Sergey S. Kiselev, Alexander A. Simenel

**Affiliations:** 1 A.N. Nesmeyanov Institute of Organoelement Compounds, Russian Academy of Sciences, 28 Vavilov St., 119991 Moscow, Russia; yuaborisov@mail.ru (Y.A.B.); kuzmenko@mail.ru (Y.V.K.); davank@ineos.ac.ru (V.A.D.); mikhail-ilin2007@yandex.ru (M.M.I.); kotosok@yandex.ru (M.M.I.J.); arhipov@mail.ru (D.E.A.); alex@xrlab.ineos.ac.ru (A.A.K.); kiss@ineos.ac.ru (S.S.K.); alexsim@ineos.ac.ru (A.A.S.); 2 N.I. Pirogov Russian National Research Medical University, 1 Ostrovityanov St., 117997 Moscow, Russia; 3 Moscow Institute of Steel and Alloys National University of Science and Technology, 4 Leninskii Av., 119049 Moscow, Russia

**Keywords:** ferrocene, pyrazoles, enantioenriched compounds, enantiomers, X-ray crystallography, HPLC, cyclodextrin, quantum chemical calculations

## Abstract

Enantiomeric-enriched ferrocene-modified pyrazoles were synthesized via the reaction of the ferrocene alcohol, (*S*)-FcCH(OH)CH_3_ (Fc = ferrocenyl), with various pyrazoles in acidic conditions at room temperature within several minutes. X-ray structural data for racemic (*R*,*S*)-1*N*-(3,5-dimethyl pyrazolyl)ethyl ferrocene (**1**) and its (*S*)-enantiomer (*S*)-**1** were determined. A series of racemic pyrazolylalkyl ferrocenes was separated into enantiomers by analytical HPLC on β- and γ-cyclodextrins (CD) chiral stationary phases. The quantum chemical calculations of interaction energies of β-CD were carried out for both (*R*)- and (*S*)-enantiomers. A high correlation between experimental HPLC data and calculated interaction energies values was obtained.

## 1. Introduction

Enantioselective synthesis is a key process in modern chemistry and is particularly important in the field of medicinal chemistry, as the different enantiomers or diastereomers of a molecule often have different biological activities. The thalidomide tragedy in the early 1960s led to much stricter testing requirements for drugs and pesticides before they can be licensed. It is especially noted into Pharmacopoeias of many countries or regions [1,2].

The unique properties of organometallic compounds, intermediates between those of classical inorganic and organic materials, provide new opportunities in medicinal chemistry. A broad variety of ferrocene compounds are regarded as potential drug candidates for medical treatments [3,4,5,6,7,8]. Such diseases as anemia and ozena can be readily cured with the help of ferrocerone, the first ferrocene-based drug [1,4,9,10,11]. Many research groups investigated different ferrocene compounds against malaria, tuberculosis, and particularly cancer [12,13,14,15,16,17,18,19,20]. The results of electrophysiological in vivo experiments with ferrocenepyrazolyl-based glycine carried out in the CA1 field of the hippocampus have recently been reported [21].

Efficient synthetic approaches towards ferrocene modifications of organic compounds have been summarized and analyzed in the recent reviews [22,23]. For preparing ferrocene-based chiral complexes, multi-step asymmetric synthesizes must be carried out often [24]. This access to enantiomeric-enriched ferrocene products was well-studied by I. Ugi and co-workers in the early 1970s [25,26,27]. To our surprise, biological research upon chiral ferrocene-based individual stereo isomers has not been realized in detail, excluding the analysis of the effects of ferrocene-based compound named ferroquine, as an antimalarial agent [28], ferrocene-hormones for specific receptors [29], and ferrocene-thymine conjugates, which can be used as anticancer agents [30]; all investigated ferrocene compounds have planar chirality.

**Scheme 1:**
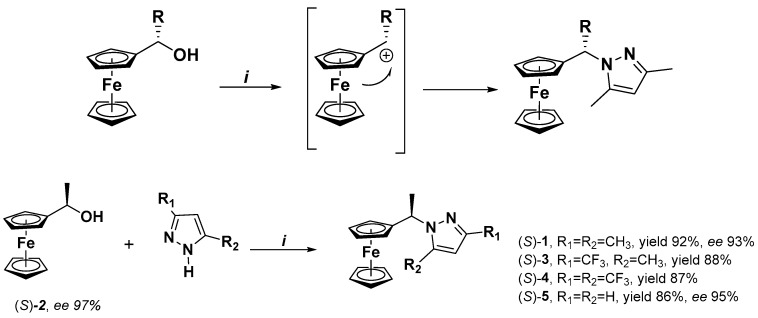
Enantiospecific synthesis of (*S*)-ferrocenylethylpyrazoles. (*i*) Methylene dichloride, HBF_4_ 45% water solution, 3,5-dimethylpyrazole (R_1_ = R_2_ = CH_3_), or pyrazoles (R_1_ = R_2_ = H), 3-trifluoromethyl-5-methylpyrazole (R_1_ = CF_3_, R_2_ = CH_3_), 3,5-di(trifluoromethyl)pyrazoles (R_1_ = R_2_ = CF_3_), 22–25 °C, 5 min; ascorbic acid (5–10 mg) was added during the work-up of the product.

In this paper, the development of our ferrocene-devoted works [31,32,33,34,35,36] are reported. We proposed a simple route to enantiomeric-enriched ferrocene-based compounds. The stereogenic center in α-position to the ferrocene moiety, where stabilization by electron-saturated ferrocene occurs, was used for chiral syntheses by means of the substitution reaction through chiral α-ferrocenylalkyl cations (Scheme 1).

Enantiomeric-enriched (*S*)-ferrocene-modified pyrazoles were synthesized via the reaction of the enantioenriched ferrocene alcohol (*S*)-FcCH(OH)Me (Fc = ferrocenyl) with pyrazoles in acidic conditions according to the approach previously developed by us for racemic ferrocene heterocycles [17,31,32]. X-ray structural data for racemic 1*N*-(3.5-dimethyl pyrazolyl)ethyl ferrocene (**1**) and its (*S*)-enantiomer (*S*)-**1** were obtained. Using a combination of single-crystal X-ray diffraction data and a chiral analytical HPLC technique, (*S*)-ferrocene pyrazoles were characterized. A series of racemic (*S,R*)-ferrocene pyrazoles was separated into enantiomers by analytical HLPC on chiral β- or γ-cyclodextrins. Quantum chemical calculations of interaction energies of (*R*)- and (*S*)-enantiomers of ferrocene conjugates with β-cyclodextrin (β-CD) chiral selector were carried out. A high correlation between calculated interaction energies (Δ*E*) and experimental HPLC data (separation factor, α) was obtained.

## 2. Results and Discussion

### 2.1. Synthesis

The ferrocenylalkylation method for the introduction of ferrocenylalkyl groups into various nucleophilic substrates was based on the reaction of α-(hydroxy)alkyl ferrocenes or ferrocenylalkyl amines with nucleophiles [36].

Imidazoles and pyrazoles, being the central ingredient in many drugs, are often used for chemical modification by ferrocene for medicinal investigations [22,23,36,37]. Usually the design and synthesis of ferrocene–heterocycle derivatives either in racemic forms or without stereogenic centers are described. As initial compounds, ferrocenylalkyl amines and Fc-alcohols are used for the synthesis of target substances [38,39,40,41,42,43,44].

As for enantioenriched ferrocene compounds, this area is much less investigated [45]. Direct enantiospecific substitution of ferrocene alcohols via acid-catalyzed C-O bond cleavage gives a variety of ferrocene-modified heterocycles [46]. Recently, it was shown that in the presence of ZnCl_2_ enantioenriched α-aminoethyl ferrocene, FcCH(CH_3_)NH_2_ (96% *ee*), reacted with *N*-methylindole and parent compounds for 6 h in acetonitrile without extrusion of air and moisture at 100 °C, gave the substitution products in excellent yields and with complete retention of configuration [47]. Much earlier, A. Togni and co-workers performed the preparation of a great variety of chiral ferrocenes, incorporating a pyrazole and phosphine by the substitution reaction in acetic acid at 70–90 °C for 3–7 h with configuration retention at the stereogenic center of initial phosphinoferrocenyl amine derivatives [48].

It is known that ferrocene alcohols as racemates, such as (*R*,*S*)-FcCH(OH)R, in acidic conditions, form highly kinetic active ferrocenyl carbocations, such as FcC^+^HR [49]. Such primary, secondary, and tertiary Fc-carbocations as solids were firstly synthesized and characterized by NMR [50] and later X-ray analysis data for permethylated ferrocene carbocation, and (C_5_Me_5_)Fe(C_5_Me_4_)C^+^H_2_ was reported [51]. Moreover, the density-functional method was used for ab initio calculations of the geometry and total energy of (C_5_Me_5_)Fe(C_5_Me_4_)CH_2_-cation [52]. Chiral FcC^+^HR are known and have been extensively used to access various chiral ferrocene derivatives [25,27]. If the stabilization effects of the solvents are not significant, Fc-carbocations prepared in situ can easily react with different nucleophiles, in particular azoles to form ferrocenylalkyl azoles, such as FcCH(R)Az [22]. This idea was successfully used for stereospecific synthesis here.

In this paper, a simple method for enantioenriched ferrocenylalkyl pyrazoles was developed, and the stereochemical aspects of ferrocene-containing pyrazoles were studied. We found that (*R*)- and (*S*)-ferrocene alcohols, when interacting with pyrazoles in aqueous-organic media in the presence of strong inorganic acid HBF_4_, give the corresponding pyrazole-containing ferrocene products, i.e., FcCH(R)-Pz, in enantiomeric-enriched forms (enantiomeric excess more than 90%, HPLC data). The complete absence of inversion or racemization at these reaction conditions makes this process a valuable preparative method for enantioenriched ferrocene-modified compounds. The stabilization of the carbocation center by electron-rich ferrocene fragment governs the stereoselectivity of the reactions. This effective strategy has been previously applied for the synthesis of racemic Fc-azoles and Fc-nucleic bases [15,18,22,32,35,53].

Herein, the one-pot synthesis of enantioenriched ferrocenylethyl 3,5-dimethylpyrazole (*S*)-**1** and other substituted pyrazoles (*S*)-**3**, (*S*)-**4** as well as an unsubstituted pyrazole (*S*)-**5** was realized (Table 1). By using (*S*)-1-ferrocenylethanol and pyrazole, 3,5-dimethylpyrazole (or fluoro-containing pyrazoles) and strong acid aqueous HBF_4_ in a molar ratio of 1:1:1 in methylene dichloride, and after stirring for a short time at room temperature, the products were synthesized in excellent yields (Scheme 1). It should be noted that to prevent the oxidation of the final product during the work-up, ascorbic acid was used. In Table 1 the optical rotation values and enantiomeric excess (HPLC data) are given for compounds (*S*)-**1**−(*S*)-**5**.

Initial (*S*)-1-ferrocenylethanol (*S*)-**2** was synthesized from acetylferrocene following the usually well-reproduced protocol developed by Ugi and co-workers, with some modifications including a series of chemical transformations [26,27]. According to the analytical HPLC data, 97% enantiomeric excess (*ee*) was achieved (see Table 1). It should be noted that enantioenriched 1-ferrocenylethanols can be prepared by the catalytic reduction of acetylferrocene with good chemical yields and *ee* [54,55]. However, (*R*)- and (*S*)-Fc-ethanoles are accessible via Ugi’s classical method. This is the more preferred synthetic route for our future biological studies as the subsequent medicinal trials demand both (*R*)- and (*S*)-enantiomers, in accordance with Russian Federation Pharmacopoeia [1]. In spite of the fact that modern ferrocene chemistry is extensively developing, especially in the field of medicine, biological studies of enantioenriched ferrocene compounds are currently limited [28,29,30].

### 2.2. Enantiomeric Resolution

In this section, racemic ferrocenylethyl pyrazoles were considered. These ferrocene-containing enantiomers with central chirality were resolved on β- or γ-cyclodextrins as chiral selectors using the HPLC analytical method. Earlier, this method of separation was successfully applied for enantiomeric ferrocene compounds carrying different simple substituents [56], as well as for ferrocenylalkyl azoles [57,58] and mercapto benzimidazoles [19], ferrocene-modified pyrimidines [18], and thiopyrimidines [35].

The enantiomeric resolution analytical data are summarized in Table 2. We successfully separated the five pairs of investigated compounds **1**, **2** and **5**–**7**. Under the conditions applied, there was no enantiomeric resolution of compounds **3** and **4**. The recognition mechanism on β-CD and γ-CD is apparently connected with the formation of specific hydrogen bonds between the strongly basic nitrogen atoms of the corresponding pyrazole fragments and the hydrogen atoms of the hydroxyl on the cavity of cyclodextrins, as in the case of simple ferrocene derivatives such as ferrocenylethanol [56] or ferrocenylalkyl azoles [53,57].

Chiral centers in the investigated compounds are represented either by the carbon atom connecting a ferrocene moiety with a heterocycle (**1**–**5**), or they are introduced in the substituent of a heterocycle (**6** and **7**). Experimental data for compounds **6** and **7** were published earlier [53]. The most efficient HPLC separation was achieved in the case of compounds **6** and **7**, with polyfluoro-containing benzimidazoles substituents, α = 1.308 for **6** and α = 1.385 for **7** (Table 2).

### 2.3. Calculations

**Figure 1:**
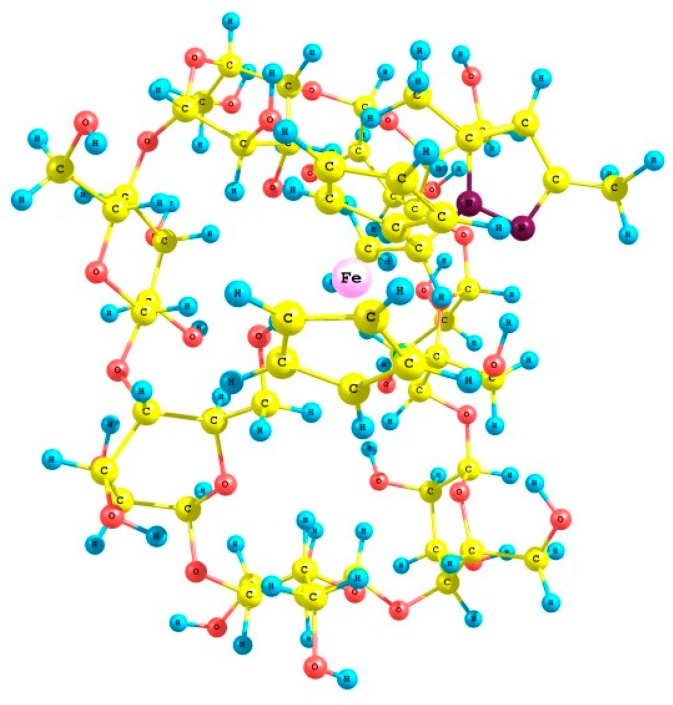
Complex (*R*)-(3,5-dimethylpyrazolyl)-α-ethyl ferrrocene CD-cyclodextrin (calculated data).

**Figure 2:**
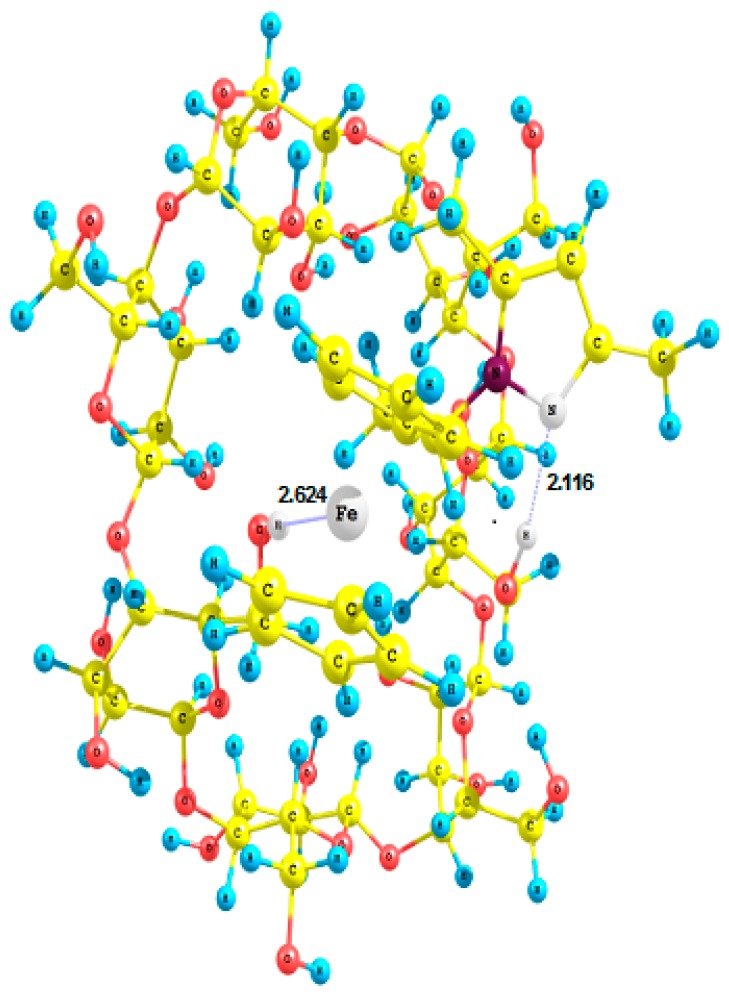
Complex (*S*)-(3,5-dimethylpyrazolyl)-α-ethyl ferrocene-CD-cyclodextrin (calculated data).

Figure 1 and Figure 2 show the structures of β-CD complexes with (*R*)- and (*S*)-enantiomers, received as the result of energy optimization (top view β-CD). Attached molecules of enantiomeric 1*N*-(3,5-dimethylpyrazolyl)ethyl ferrocene are situated over the torus β-CD.

Table 3 shows the calculated characteristics of β-CD complexes with (*R*)- and (*S*)-enantiomers, including the comprehensive energy from the calculation of the DFT B3LYP/LanL2DZ, the computed values of dipole moments, and the highest occupied molecular orbital (HOMO) and lowest unoccupied molecular orbital (LUMO) energies (a.u.—atomic units).

**Figure 3:**
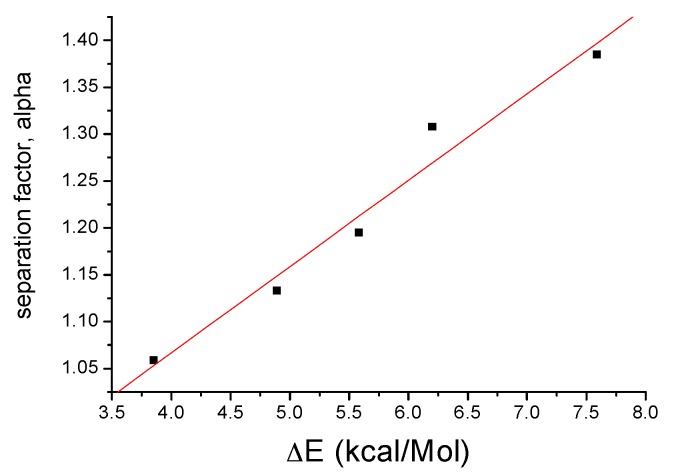
Correlation between experimental HPLC data (alpha) and Δ*E* calculated interaction energies values. α = 0.69862 + 0.09203 Δ*E*. The multiplier at Δ*E* is a tangent of the angle inclination of a line.

In Table 2, the experimental analytical HPLC data and the calculated Δ*E* energies for β-CD complexes with 1*N*-(3,5-dimethylpyrazolyl)ethylferrocene (*R*)- and (*S*)-enantiomers as well as other Fc-compounds are summarized, including ferrocene-based bezimidazoles **6** and **7**. Note that we failed in resolving fluoro-containing pyrazoleferrocenes **3** and **4** on chiral cyclodextrins. Rather strong intermolecular hydrogen bonds probably complicate this process. The red-marked values for these compounds at α = 1.153 and α = 1.182 (Table 2) were accordingly defined from the linear correlation (Figure 3).

The interaction energy of (*S*)-enantiomer 1*N*-(3,5-dimethyl pyrazolyl) ethyl ferrocene (*S*)-**1** with β-CD is 3.85 kcal mol^−1^ higher than the interaction energy of the *(R)*-enantiomer with β-CD, as it was calculated. Thus, complex β-CD-(*S)*-Fc-enantiomer is more stable than complex β-CD-(*R)*-Fc-enantiomer. The reason for such differences in energies of interaction, Δ*E,* is that the (*R)*-enantiomer-containing complex has only one H-bond connecting the *N*-heterocyclic atom with the OH-function of β-CD. The bond length N…H amounts to 2.057 Å (Figure 1). The complex of β-CD with (*S*)-Fc-enantiomer has two H-bonds. One bond is formed between the *N*-pyrazole atom of pyridine type and the OH-function of β-CD. The bond length amounts to 2.116 Å. Another H-bond connects the Fe-atom with the OH-function of β-CD. The bond length Fe…H is 2.624 Å. These bonds are marked on Figure 2. These three H-bonds are rather weak [59,60], and therefore racemates with small Δ*E* energies were easily separated on cyclodextrins.

Quantum chemical calculations of hydrogen bonds and energies were made for (*R*)-, (*S*)-enantiomers and β-cyclodextrins as chiral selectors. The data obtained demonstrate the high correlation between the experimental retention factors and the calculated interaction energies (Figure 3).

In conclusion, the combined calculation data and experimental racemic resolution data could be useful for the determination of the absolute configuration of compounds.

### 2.4. Crystal Structures

**Figure 4:**
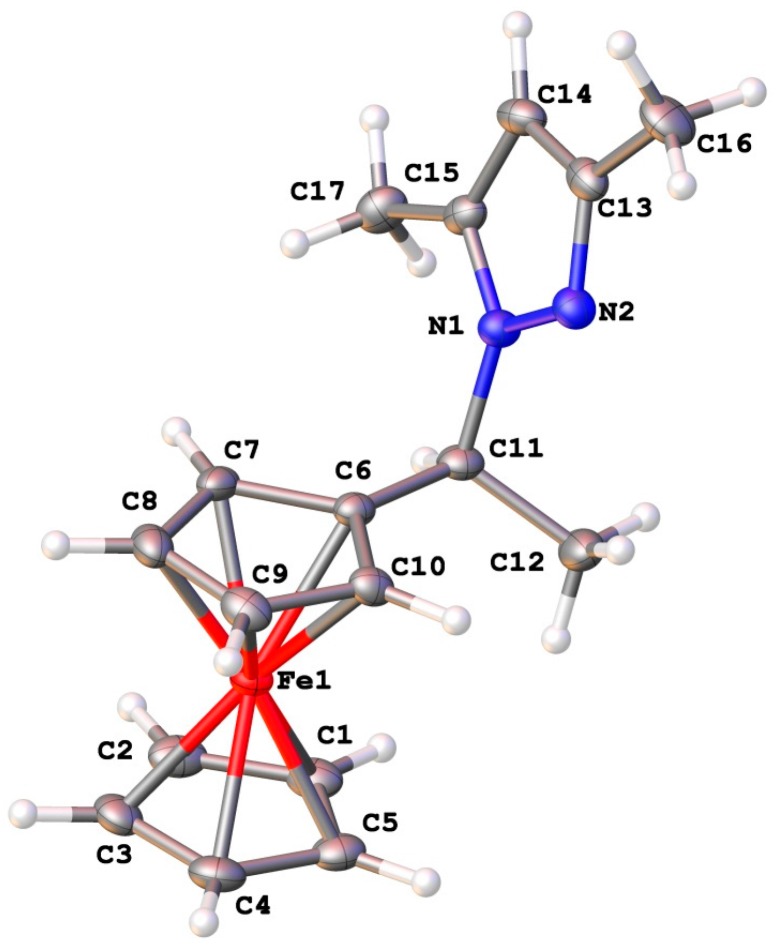
Molecular structure of **1** presented in thermal ellipsoids at 50% probability. Selected lengths, Å, and angles (°), C6-C11 1.509(4); N1-C11 1.470(3); C11-C12 1.527(4); Fe1-C6 2.040(2); N1-N2 1.366(3); N1-C11-C6 110.5(2); N1-C11-C12 109.5(2); C7-C6-C11 126.4(2); C6-C11-C12 113.1(2).

We proceeded to crystallize the racemic 1*N*-(3,5-dimethyl pyrazolyl)ethyl ferrocene (**1**) and its (*S*)-enantiomer (*S*)-**1**. The molecular structure of **1** is shown in Figure 4. It was found that the domain structure of **1** consists of blocks containing both the (*S*)- and (*R*)-enantiomers in a 1:1 ratio. The structures of **1** and (*S*)-**1** are slightly divers due to various experimental conditions. However, the discrepancies in the values of bond lengths and angles are negligible.

The value of bond lengths and angles fall in the same range as the bond lengths and angles in the ferrocene derivatives bearing heterocyclic fragments [18,19,31,32,33,53]. The cyclopentadienyl ligands are in the eclipsed conformation that is rather typical for substituted ferrocenes [31,32,33]. The C2-Ct1-Ct2-C7 angles are equal to 2.6(2) and 2.7(5) (Ct1 and Ct2 are the centroids of the Cp rings). The molecular structure of **1** in crystal is characterized by the bending of the pyrazole group with respect to the ferrocenyl moiety. The angle between the mean planes of these fragments is 74.2°.

The absolute configuration definition for compound (*S*)-**1** was made using the Flack parameter; K = −0.02 and was assigned as (*S*).

## 3. Experimental Section

### 3.1. Methods and Materials

^1^H- and ^13^C-NMR spectra were obtained on a Bruker DRX-500 spectrometer (Bruker Billerica, MA, USA) at 500.13 MHz and 125.76 MHz for protons and ^13^C, respectively, in CDCl_3_ at 30 °C. Chemical shifts are given in ppm relative to solvent residual protons. IR spectra were recorded on a UR-20 spectrophotometer (Carl Zeiss, Jena, Germany), using a KBr disk. Electron ionization (EI) mass spectra were taken on a FINNIGAN POLARIS Q spectrometer (Thermo Fisher Scientific, Waltham, UK) at 70 eV, with the temperature of the ion chamber at 250 °C. The solvents were purified by standard techniques. The following chiral columns (250 × 4.6 mm, 5 µm) were used: β-cyclodextrin (Cyclobond I 2000), (*S*)-naphthylcarbamate-derivatized-β-cyclodextrin (Cyclobond I 2000 SN). Chromatographic resolution was carried out on an HPLC system (Advanced Separation Thechnologies, Inc., Whippany, NJ, USA), with a Bruker LC 31 instrument equipped with a UV detector (254 nm); the flow rate was 1.0 mL·min^−1^ at an ambient temperature. The specific rotation was determined on a Perkin-Elmer 141 polarimeter.

1-(*R*,*S*)-Ferrocenylethanol was synthesized by the reduction of acetylferrocene with LiAlH_4_ in THF according to a modified well-known procedure [53,61]. Initial (*S*)-*N,N*-dimethylaminoethylferrocene for the synthesis of (*S*)-1-ferrocenylethanol was obtained by the crystallization of racemic amine with *L*-tartaric acid [26], and was then transformed into (*S*)-1-ferrocenylethanol [27]. The synthesis of ferrocenylalkyl azoles both in racemic and enantiomeric-enriched forms was carried out by previously described methods [31,33,53]. Initial heterocycles were purchased from Acros Organics and used without purification. The experimental data for enantiomeric-enriched compounds are summarized in Table 1.

### 3.2. General Procedure

To a mixture of 1.0 mmol, 0.23 g of (*S*)-1-ferrocenylethanol, *ee* 97% (HPLC data), [α]${}_{D}^{20}$ = +30.75, 0.60 mol·L^−1^ (methanol), 1.0 mmol of the corresponding heterocycle in 1.0 mL of methylene dichloride, and 0.18 mL of 45% aqueous solution of fluoroboric acid was added under vigorous stirring. The agitation was continued for 5 min, and then Et_2_O (15 mL), the same amount of cold water, and 5–10 mg of ascorbic acid were added to the reaction flask. After vigorous shaking of the mixture, the organic solution was separated, washed with cold water, the solvent was removed, and the residue was dried over CaCl_2_ in a desiccator.

*(S)-(3,5-Dimethylpyrazolyl)-α-ethylferrrocene* (*S*)-**1**, yield 92%, 0.28 g, *ee* 93%, orange crystals, m.p. 76 °C, ^1^H-NMR (CDCl_3_, δ, ppm): 1.83 (d, *J* = 6.8 Hz, 3H, CH_3_); 2.20 (s, 3H, CH_3_); 2.23 (s, 3H, CH_3_); 4.13 (s, 2H, Fc); 4.15 (s, 5H, Fc); 4.20 (s, 1H, Fc); 4.22 (s, 1H, Fc); 5.30 (q, *J* = 6.8 Hz, 1H, CH); 5.75 (s, 1H, (C-4)Pz). ^13^C-NMR (CDCl_3_, δ, ppm): 11.6 (CH_3_), 13.7 (CH_3_), 20.2 (CH_3_), 53.9 (CH), 66.7 (C_5_H_4_), 67.3 (C_5_H_4_), 67.8 (C_5_H_4_), 68.3 (C_5_H_4_), 68.8 (C_5_H_5_), 89.9 (ipso-C_5_H_4_), 105.2 (Pz, C-4), 137.7 (Pz, C-5), 146.6 (Pz, C-3). IR (KBr, ν, cm^−1^): 3010, 2890, 1560, 1470, 1120, 1009, 840, 778, 539, 472. EI-MS, *m*/*z* (RI, %): 308 [M]^+^ (92).

*(S)-(3-Trifluoromethyl-5-methylpyrazolyl)-α-ethylferrrocene* (*S*)-**3**, yield 88%, 0.32 g, yellow crystals, m.p. 47 °C, ^1^H-NMR (CDCl_3_, δ, ppm): 1.85 (d, *J* = 9.6 Hz, 3H, CH_3_); 2.25 (s, 3H, CH_3_); 4.04 (s, 1H, Fc); 4.13 (m, 7H, Fc); 4.21 (s, 1H, Fc); 5.33 (q, *J* = 9.6 Hz, 1H, CH); 6.23 (s, 1H, (C-4)Pz). ^13^C-NMR (CDCl_3_, δ, ppm): 11.5 (CH_3_), 20.4 (CH_3_), 55.0 (CH), 66.5 (C_5_H_4_), 67.4 (C_5_H_4_), 67.8 (C_5_H_4_), 68.4 (C_5_H_4_), 68.9 (C_5_H_5_), 89.3 (*ipso*-C_5_H_4_), 103.7 (q, *J* (C-^3^F) = 1.8 Hz, Pz, C-4), 124.4 (q, *J* (C-F) = 266.7 Hz, CF_3_), 138.8 (Pz, C-5), 140.5 (q, *J* (C-^2^F) = 37.5 Hz, Pz, C-3). IR (KBr, ν, cm^−1^): 3029, 2898, 1564, 1456, 1265, 1120, 1011, 844, 776, 547, 472. EI-MS, *m*/*z* (RI, %): 362 [M]^+^ (89).

*(S)-(3,5-Ditrifluoromethylpyrazolyl)-α-ethylferrrocene* (*S*)-**4**, yield 87%, 0.36 g, orange oil, ^1^H-NMR (CDCl_3_, δ, ppm): 1.88 (d, *J* = 9.6 Hz, 3H, CH_3_); 4.14–4.22 (m, 9H, Fc); 5.45 (q, *J* = 9.6 Hz, 1H, CH); 6.56 (s, 1H, (C-4)Pz). ^13^C-NMR (CDCl_3_, δ, ppm): 20.6 (CH_3_), 58.1 (CH), 66.1 (C_5_H_4_), 67.0 (C_5_H_4_), 67.7 (C_5_H_4_), 68.3 (C_5_H_4_), 68.9 (C_5_H_5_), 89.6 (*ipso*-C_5_H_4_), 106.3 (m, Pz, C-4), 119.8 (q, *J* (C-F) = 267 Hz, CF_3_), 122.4 (q, *J* (C-F) = 267 Hz, CF_3_), 138.2 (q, *J* (C-^2^F) = 37.5 Hz, Pz, C-5), 141.4 (q, *J* (C-^2^F) = 37.5 Hz, Pz, C-3). IR (KBr, ν, cm^−1^): 3010, 2964, 1575, 1510, 1462, 1415, 1396, 1163, 1040, 987, 840, 715, 547, 498. EI-MS, *m*/*z* (RI, %): 416 [M]^+^ (100).

*(S)-(Pyrazolyl)-α-ethylferrrocene* (*S*)-**5**, yield 86%, 0.24 g, *ee* 95%, orange crystals, m.p. 54 °C, ^1^H-NMR (CDCl_3_, δ, ppm): 1.86 (d, *J* = 6.4 Hz, 3H, CH_3_); 4.17–4.29 (m, 9H, Fc); 5.41 (q, *J* = 6.8 Hz, 1H, CH); 6.21 (s, 1H, (C-4)Pz); 7.30 (s, 1H, Pz); 7.51 (s, 1H, (C-4)Pz). ^13^C-NMR (CDCl_3_, δ, ppm): 21.4 (CH_3_), 57.3 (CH), 66.2 (C_5_H_4_), 67.9 (C_5_H_4_), 68.0 (C_5_H_4_), 68.5 (C_5_H_4_), 68.9 (C_5_H_5_), 89.0 (ipso-C_5_H_4_), 105.0 (Pz, C-4), 126.7 (Pz), 138.4 (Pz, C-3). IR (KBr, ν, cm^−1^): 3115, 2965, 1525, 1411, 1293, 1120, 1045, 968, 853, 720, 665, 538, 498. EI-MS, *m*/*z* (RI, %): 280 [M]^+^ (82).

### 3.3. Calculations

The calculations of β-cyclodextrin (β-CD), 1*N*-(3,5-dimethyl pyrazolyl)ethyl ferrocene (*R*)- and (*S*)-enantiomers as well as the interaction energies of β-CD with (*R*)- and (*S*)-enantiomers were performed by the restricted Becke−Lee−Young−Parr modification (RB3LYP) of the density functional theory [62,63,64]. The Dunning−Hay D95 atomic basis sets were used for the optimization of the geometry and for the calculation of the interaction energies between β-CD and *R*- and *S*-enantiomers.

### 3.4. Crystallography

Single crystals suitable for X-ray diffraction analysis were obtained by recrystallization from acetone-*d*_6_ compounds **1** and (*S*)-**1**. X-ray diffraction measurements were carried out using CAD4 Enraf-Nonius and Bruker Smart APEX II diffractometers. The structures were solved by a direct method and refined by the full-matrix least-squares technique against F^2^ in an anisotropic approximation. Hydrogen atoms were located from the difference Fourier maps and refined in rigid body model. All calculations were performed using the SHELXTL program package [65]. Details of crystallographic data and experimental conditions are presented in Table 4.

Bond lengths and angles, thermal parameters, and experimental conditions of the structures **1** and (*S*)-**1** have been deposited in the Cambridge Crystallographic Data Centre (CCDC Nos. 1491349 and 1491350) and can be obtained free of charge via the webpage: http://www.ccdc.cam.ac.uk/Community/Requestastructure/pages/Requestastructure.aspx.

## 4. Conclusions

In summary, we reported an efficient, highly enantiospecific synthesis of chiral pyrazole ferrocenes via direct substitution under acidic conditions in two-phase media from ferrocene alcohols and pyrazoles in good yields with very high enantiomeric purity.

## Figures and Tables

**Table 1:** Enantiomeric excess and optical rotation of ferrocene compounds (for formulas see Table 2).

No	Molecule	Enantiomeric Excess, *ee* % ^(a)^	[α]${}_{D}^{20}$	Concentration, mole·L^−1^
(*S*)-**1**	FcCH(CH_3_)-3,5-Me_2_Pz	93	+78.75	0.40 (benzene)
(*S*)-**2**	FcCH(CH_3_)OH	97	+30.75	0.60 (methanol)
(*S*)-**3**	FcCH(CH_3_)-3-CF_3_,5-CH_3_ Pz	not determined	+12.00	0.30 (benzene)
(*S*)-**4**	FcCH(CH_3_)-3-CF_3_,5-CF_3_ Pz	not determined	+14.30	0.18 (benzene)
(*S*)-**5**	FcCH(CH_3_)Pz	95	+16.50	0.70 (benzene)

^(a)^ Found by HPLC method.

**Table 2:** Enantiomeric resolution of racemic mixtures **1**–**7** on column Cyclobond I 200 and calculated data for selectivity energies.

No	Molecule ^(a)^	Chiral Stationary Phase ^(b)^	Separation Factor, α	Δ*E*, kcal/*M*
**1**	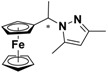 FcCH(CH_3_)-3,5-Me_2_Pz	β-CD	1.059	3.85
**2**	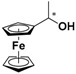 FcCH(CH_3_)OH	β-CD	1.133	4.89
**3**	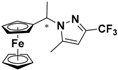 FcCH(CH_3_)-3-CF_3_,5-CH_3_Pz	β-CD	1.153	4.94
**4**	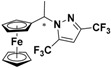 FcCH(CH_3_)-3-CF_3_,5-CF_3_Pz	β-CD	1.182	5.25
**5**	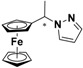 FcCH(CH_3_)Pz	β-CD (c)	1.195	5.58
**6**	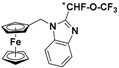	γ-CD (d)	1.308	6.20
**7**	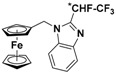	γ-CD (d)	1.385	7.59

^(a)^ ∗ in the structures means the stereogenic center; ^(b)^ Mobile phase, hexane–isopropanol 98:4 (*v/v*); ^(c)^ For compound **5**, experimental data were found on modified cyclodextrin, column Cyclobond I 200 SN; ^(d)^ For compounds **6** and **7**, see experimental details in Reference [53].

**Table 3:** Calculated characteristics of complexes β-CD with *R*- and *S*-enantiomers.

System	*E*, a.u.	μ, D	HOMO, a.u.	LUMO, a.u.
(*R*)-1-β-CD	−5167.4437	4.25	−0.19354	−0.02009
(*S*)-1-β-CD	−5167.4481	6.25	−0.20897	−0.03714

**Table 4:** Crystallographic data and refinement parameters for the structures **1** and (*S*)-**1**.

	1	(*S*)-1
Diffractometer	Bruker Smart APEX II	CAD4 Enraf-Nonius
T, K	120	293
Empirical formula	C_17_H_20_FeN_2_	C_17_H_20_FeN_2_
Formula weight	308.20	308.20
Crystal system	Orthorhombic	Orthorhombic
Space group, *Z*	*P2_1_2_1_2_1_*, 4	*P2_1_2_1_2_1_*, 4
Density_calc._ (g·cm^−3^)	1.396	1.346
*a* (Å)	7.8662(5)	7.9670(16)
*b* (Å)	8.9661(5)	8.9670(18)
*c* (Å)	20.7985(13)	21.291(4)
*V* (Å^3^)	1466.90(15)	1521.0(5)
2*θ*_max_ (°)	60.12	49.91
F(000)	648	648
Reflections collected	19,371	1649
Independent reflections (*R*_(int)_)	4301 (0.0542)	1565 (0.0108)
Number of reflections with *I* > 2*σ*(*I*)	3766	1364
Parameters	185	184
Flack	0.467(18)	−0.02(4)
Linear absorption (cm^−1^)	10.19	9.83
Goodness-of-fit (GOF)	1.010	0.998
*R*_1_ (*I* > 2*σ*(*I*))	0.0330	0.0396
*wR*_2_ (all reflections)	0.0698	0.1054
*ρ*_min_/*ρ*_max_, e	0.338/−0.397	0.366/−0.661
